# Evaluating the Impact of DNA Extraction Method on the Representation of Human Oral Bacterial and Fungal Communities

**DOI:** 10.1371/journal.pone.0169877

**Published:** 2017-01-18

**Authors:** Anna Vesty, Kristi Biswas, Michael W. Taylor, Kim Gear, Richard G. Douglas

**Affiliations:** 1 Department of Surgery, The University of Auckland, Auckland, New Zealand; 2 School of Biological Sciences, The University of Auckland, Auckland, New Zealand; 3 Oral Health Unit, Green Lane Clinical Centre, Auckland, New Zealand; Columbia University, UNITED STATES

## Abstract

The application of high-throughput, next-generation sequencing technologies has greatly improved our understanding of the human oral microbiome. While deciphering this diverse microbial community using such approaches is more accurate than traditional culture-based methods, experimental bias introduced during critical steps such as DNA extraction may compromise the results obtained. Here, we systematically evaluate four commonly used microbial DNA extraction methods (MoBio PowerSoil^®^ DNA Isolation Kit, QIAamp^®^ DNA Mini Kit, Zymo Bacterial/Fungal DNA Mini Prep^TM^, phenol:chloroform-based DNA isolation) based on the following criteria: DNA quality and yield, and microbial community structure based on Illumina amplicon sequencing of the V3–V4 region of the 16S rRNA gene of bacteria and the internal transcribed spacer (ITS) 1 region of fungi. Our results indicate that DNA quality and yield varied significantly with DNA extraction method. Representation of bacterial genera in plaque and saliva samples did not significantly differ across DNA extraction methods and DNA extraction method showed no effect on the recovery of fungal genera from plaque. By contrast, fungal diversity from saliva was affected by DNA extraction method, suggesting that not all protocols are suitable to study the salivary mycobiome.

## Introduction

The oral cavity harbours one of the most diverse microbiomes in the human body [1]. Within the oral cavity, several distinct niches occur, including those found in plaque and saliva [2, 3], where dysbiosis and the presence of specific microbes can be associated with disease [4–6]. Choice of DNA extraction protocol has the potential to influence our perception of microbiome structure. DNA extraction is achievable via different cell lysis procedures, including chemical, enzymatic, mechanical and heat. Recent studies demonstrate that the cell lysis method used during DNA extraction from oral samples can impact the recovery of specific bacterial phyla [7]. Mechanical lysis increases the number of different bacterial phyla recovered from saliva [7], while the addition of lysozyme to mechanical lysis improves overall bacterial DNA yield from saliva [8]. Similarly, in plaque a mechanical lysis step and the addition of lysozyme maximises recovery at the species level [9]. As all methods have inherent biases, an approach that provides a true representation of the oral microbiome is vital if we are to more fully understand its clinical manifestations.

Studies of DNA extraction bias have largely focused on the bacterial constituents of the oral microbiome, thus data pertaining to the fungal community (mycobiome) are lacking by comparison. Identical host (human) and fungal sequences at 18S rRNA gene primer binding sites render this gene an unsuitable target for many human samples, as sequences obtained may be predominantly human- rather than fungal-derived. Partly for this reason, the intergenic internal transcribed spacer (ITS) region has become an attractive alternative to 18S rRNA gene sequencing for fungal community analyses, due to its greater sequence variability which differentiates it from the host DNA and also allows for greater taxonomic resolution [10]. However, the advantages of ITS sequencing have not yet led to a greatly improved understanding of the oral mycobiome. To date, only a handful of studies have attempted to describe this community, with even less emphasis on how DNA extraction may affect its representation [11–13].

Physical and chemical interactions occur between bacteria and fungi in the oral environment, driving the structure and behaviour of the oral microbial community and potentially contributing to the pathogenesis of oral diseases [14–16]. Hence, it would be advantageous to simultaneously study DNA from both bacterial and fungal communities, in order to understand the clinical associations of both intra- and inter-domain relationships. Here, we systematically evaluate four genomic DNA (gDNA) extraction methods in order to compare bacterial and fungal community profiles, and determine which methods provide a suitable representation of microbial diversity in human dental plaque and saliva samples.

## Materials and Methods

### Ethics statement

Ethical approval for this study was granted by the Southern Health and Disability Ethics Committee (HDEC) (14/STH/121) and Institutional Approval was gained from the Auckland District Health Board, New Zealand. Written consent was obtained from all participants.

### Sample collection and preparation

Twelve patients were recruited from the Oral Health Unit, Green Lane Hospital, Auckland, New Zealand to participate in this study. All patients consented to providing sub- and supra-gingival dental plaque for this study, collected during a whole mouth scale. Plaque was removed by a registered dentist using sterile periodontal scalers and placed in a screw cap tube containing 1 mL of RNA*later*^®^ (AMBION, Inc., Austin, TX, USA). Samples were stored at -20°C until further processing. Twelve healthy volunteers from the University of Auckland, New Zealand consented to providing a fresh saliva sample. At least 1 mL of saliva was collected from each participant via passive secretion into a sterile container and frozen neat at -20°C. To create a homogenous sample of adequate volume for the comparison of multiple DNA extraction methods, 1 mL of each of the 12 plaque samples in RNA*later*^®^ (AMBION, Inc., Austin, TX, USA) was pooled in a 50 mL CELLSTAR^®^ Polypropylene Tube (Greiner Bio-One) and vortexed until a consistent solution was achieved. In a similar manner, 1 mL of each of the 12 saliva samples was pooled and mixed thoroughly. The plaque and saliva pooled homogenates (totalling approximately 12 mL each) were divided separately into 200 μL aliquots for subsequent testing.

### DNA extraction

Four extraction techniques were performed in triplicate on the 200 μL aliquots of both the pooled plaque and pooled saliva homogenates. Three commonly used commercial DNA extraction kits were used as per manufacturer’s instructions, namely the MoBio PowerSoil^®^ DNA Isolation Kit (M), Qiagen QIAamp^®^ DNA Mini Kit (Q) and the Zymo Bacterial/Fungal DNA Mini Prep^TM^ (Z) (Table 1). Additionally, a previously described phenol:chloroform-based method for DNA isolation from saliva was used (P) [17]. Mechanical cell rupture was performed using a Qiagen TissueLyser II at 30 Hz for 2 x 50 s, as applicable. For each method, an extraction blank (PCR-grade water) was used to ascertain potential kit and/or reagent contamination.

**Table 1:** Summary of DNA extraction methods examined in this study.

Extraction method	Abbreviation	Lysis type	DNA isolation	Elution volume (μL)
**MoBio PowerSoil^®^ DNA Isolation Kit**	M	Mechanical	Spin column	100
**Qiagen QIAamp^®^ DNA Mini Kit**	Q	Enzymatic, heat	Spin column	200
**Zymo Bacterial/Fungal DNA Mini Prep^TM^**	Z	Mechanical	Spin column	100
**Phenol:chloroform-based DNA isolation**	P	Enzymatic, freeze-thaw	Phase separation	20

### Efficiency of fungal DNA extraction

The four DNA extraction methods were employed in triplicate to extract fungal DNA separately from 200 μL of ~7500 CFU/μL *Cryptococcus neoformans* ATCC^®^ 32045^TM^ in RNA*later*^®^ (AMBION, Inc., Austin, TX, USA) and 200 μL of ~1150 CFU/μL *Penicillium chrysogenum* ATCC^®^ 10002^TM^ in RNA*later*^®^ (AMBION, Inc., Austin, TX, USA). DNA quality and yield were measured on the NanoPhotometer^®^ N60 (IMPLEN, Inc., Westlake Village, CA, USA).

#### MoBio PowerSoil^®^ DNA isolation kit

Aliquots were thawed on ice and pipetted into a supplied PowerBead Tube. The MoBio PowerSoil^®^ DNA isolation kit employs a mechanical (bead beating) lysis step to rupture cells. Addition of the provided salt solution helped DNA bind to the silica spin column filter, and an ethanol-based solution washed the bound DNA. Finally, 100 μL of sterile elution buffer released the DNA from the spin column filter, yielding DNA for downstream applications.

#### Qiagen QIAamp^®^ DNA mini kit

After thawing on ice, aliquots were pelleted at 5000 x *g* and resuspended in the supplied lysis buffer. Cell lysis and protein digestion were achieved using a Proteinase K incubation at 56°C for 1 h. The DNA was bound to a spin column filter, then washed with 96–100% ethanol, followed by two wash buffers (supplied). The bound DNA was eluted from the spin column filter with 200 μL of the supplied elution buffer.

#### Zymo Bacterial/Fungal DNA mini prep^TM^

Thawed plaque and saliva aliquots were centrifuged at 5000 x *g* and resuspended in 200 μL of sterile phosphate buffered saline (PBS) in a ZR BashingBead^TM^ Lysis Tube. A lysis solution was added to help lyse cells during the mechanical lysis step. The supernatant of the lysed solution was filtered using a Zymo-Spin^TM^ IV Spin Filter, then DNA was bound to a Zymo-Spin^TM^ IIC Column in the presence of DNA Binding Buffer, containing 0.5% (v/v) beta-mercaptoethanol. DNA was washed, then eluted with 100 μL of DNA Elution Buffer.

#### Phenol:chloroform-based DNA isolation

This phase separation method was adapted from a previously described method for gDNA extraction from saliva [17]. TE Buffer (400 μL) was added to thawed plaque and saliva aliquots and the mixture centrifuged at 8000 x *g* for 5 min. The pellet was resuspended in TE Buffer containing lysozyme (5 mg/mL) and incubated for 1 h at 37°C. Proteinase K and 5% sodium dodecyl sulphate (SDS) were added to final respective concentrations of 2 mg/mL and 1% (v/w), then tubes were incubated at 50°C for 2 h, with shaking at 300 rpm. Nucleic acids were released from the cells with three freeze-thaw cycles of -20°C (5 min) and 65°C (3 min). Buffer-saturated phenol was added and tubes were vortexed, then centrifuged (13,000 x *g*, 4°C, 10 min). The aqueous phase was recovered, to which an equal volume of chloroform/isoamyl alcohol (24:1) was added. Tubes were vortexed and centrifuged again at 13,000 x *g*, 4°C for 10 min. The supernatant was transferred to a UV-sterilised Eppendorf tube and the nucleic acids were precipitated with 0.6 volume of isopropanol overnight at 4°C. The following day, DNA was pelleted (13,000 x *g*, 4°C, 10 min), washed with 70% ethanol and resuspended in 20 μL of sterile water.

### Evaluation of DNA quality and yield from plaque and saliva

DNA yield was determined fluorometrically using the High Sensitivity dsDNA kit (Invitrogen Co., Carlsbad, CA, USA) on the Qubit^®^ Fluorometer 1.0. Absorbance ratios were measured spectrophotometrically on the NanoDrop^®^ ND-1000 (NanoDrop Technologies Inc., Wilmington, DE, USA) to assess DNA purity: A260/280 nm for protein contamination and A260/230 nm for salt and phenol contamination. Since DNA absorbs light at 260 nm, ratios of 1.8–2.0 (for A260/280 nm) and > 1.8 (for A260/230 nm) indicated the sample was likely to be free from contamination by the respective substances. Genomic DNA (3 μL) was visualised on a 1% agarose gel (w/v) containing SYBR Safe DNA Gel Stain (Invitrogen Co., Carlsbad, CA, USA). DNA quantity and quality for each sample was further assessed using an Agilent DNA 1000 chip (Agilent Technologies, Waldbronn, Germany), which uses fragment size to assess DNA integrity and degradation.

### PCR amplification and sequencing preparation

DNA extracts were diluted in UltraPure^TM^ distilled water (Invitrogen Co., Carlsbad, CA, USA) to achieve equimolar concentrations. For each triplicate extraction and single extraction blank from the four different methods, gDNA was subjected to PCR amplification of both the bacterial 16S ribosomal RNA (16S rRNA) gene and fungal internal transcribed spacer 1 (ITS1) region.

#### 16S rRNA gene amplification

Primers 341F and 806R were used to amplify the V3 –V4 region of the 16S rRNA gene [18]. Each PCR reaction contained: 1X High Fidelity PCR Buffer, 2 mM magnesium sulphate, 0.5 mM dNTPs, 0.004X Platinum^®^ Taq DNA Polymerase High Fidelity (Invitrogen Co., Carlsbad, CA, USA), 0.2 μM 341F primer, 0.2 μM 806R, 18.9 μL PCR-grade water and 1 μL of each normalised gDNA template. For each amplification run, 1 μL *Escherichia coli* gDNA was used as a positive control and 1 μL of PCR-grade water as a negative control. PCR was performed using the following thermocycling conditions: Initial denaturation at 94°C for 3 min, followed by 32 cycles consisting of denaturation (94°C for 45 s), annealing (55°C for 45 s) and extension (72°C for 90 s), with a final extension step at 72°C for 10 min. Duplicate amplifications were performed for each reaction, then pooled to give a total volume of 50 μL. Two microlitres from each pooled PCR reaction were run on a 1% agarose gel (w/v) containing SYBR Safe DNA Gel Stain (Invitrogen Co., Carlsbad, CA, USA) and visualised under ultraviolet light.

#### ITS1 amplification

Primers ITS1F [19] and ITS2 [20] were used to amplify the ITS1 region. Each PCR reaction contained: 1X High Fidelity PCR Buffer, 2 mM magnesium sulphate, 0.5 mM dNTPs, 0.004X Platinum^®^ Taq DNA Polymerase High Fidelity (Invitrogen Co., Carlsbad, CA, USA), 0.2 μM ITS1 forward primer, 0.2 μM ITS2 reverse primer, 18.9 μL PCR-grade water and 1 μL of each DNA template. For each reaction, 1 μL *Candida albicans* gDNA was used as a positive control and 1 μL of PCR-grade water as a negative control. The following thermocycling conditions were used: Initial denaturation at 95°C for 3 min, followed by 38 cycles consisting of denaturation (94°C for 45 s), annealing (50°C for 30 s) and extension (72°C for 90 s), with a final extension step at 72°C for 10 min. Duplicate amplifications were performed for each reaction, then pooled to give a total volume of 50 μL. Two microlitres from each pooled PCR amplification were run on a 1% agarose gel (w/v) containing SYBR Safe DNA Gel Stain (Invitrogen Co., Carlsbad, CA, USA).

#### Preparation of PCR amplicons for illumina miSeq sequencing

For each of the pooled 16S rRNA gene and ITS1 amplicons, 40 μL was purified using AMPure XP magnetic beads (Beckman Coulter Inc., Beverly, MA, USA) to a final volume of 20 μL in sterile water. DNA concentration was measured for all purified samples using the High Sensitivity dsDNA kit (Invitrogen Co., Carlsbad, CA, USA) on the Qubit^®^ Fluorometer 1.0. Purified amplicons were subjected to further quality and quantity checks before sequencing on the MiSeq Illumina platform, performed by the Centre for Genomics, Proteomics and Metabolomics through New Zealand Genomics Ltd at The University of Auckland. Sequence data were uploaded to the NCBI Sequence Read Archive, under accession number SRP079075.

### Taxonomic assignment and bacterial diversity analyses

Raw sequences were processed using the UPARSE pipeline and QIIME 1.9 [21, 22]. Briefly, forward and reverse bacterial 16S rRNA reads were merged with a minimum merge length of 200 bp, then simultaneously filtered to remove singletons and chimeras in UPARSE [21]. Samples were rarefied to 6000 reads, and alpha diversity metrics were calculated (‘observed species’, Chao1, Shannon, Simpson) in QIIME 1.9 [22]. Operational taxonomic units (OTUs) were defined based on 97% sequence similarity, and taxonomy was assigned to individual OTUs through the Ribosomal Database Project (RDP) classifier using the Human Oral Microbiome Database (HOMD) [23]. Aligned FASTA sequences were used to build a phylogenetic tree using the make_phylogeny.py command in QIIME 1.9 for subsequent phylogenetic-based beta diversity measures. Dissimilarity matrices (weighted UniFrac, unweighted UniFrac and Bray-Curtis) were generated in QIIME 1.9 and visualised through multidimensional scaling (MDS) plots built in PRIMER v6 [24]. All three beta diversity measurements produced similar results, and we chose to use the unweighted UniFrac metric to compare beta diversity due to its previous success in distinguishing human microbial communities with a small sample size [25].

### Taxonomic assignment and fungal diversity analyses

Fungal ITS1 sequences were also processed using the UPARSE pipeline and QIIME 1.9 [21, 22]. In summary, forward and reverse fungal ITS1 reads were merged in the UPARSE pipeline using the USEARCH command fastq_mergepairs with a minimum merge length of 100 bp [21]. Sequences were filtered with an abundance threshold of more than four sequence counts, to exclude rare genera [13]. Samples were sub-sampled to 959 reads. Alpha diversity was estimated in QIIME 1.9 using the ‘observed species’, Chao1, Shannon and Simpson diversity metrics [22]. Taxonomy was assigned using the RDP classifier with UNITE OTUs v12_11 [26] as the database using a 97% sequence similarity threshold, based on 75% of fungal species containing ≤ 3% ITS1 intraspecific variation [10].

### Statistical analyses of DNA quality and yield

To compare qualitative and quantitative data from DNA extractions across the four methods, a one-way analysis of variance (ANOVA) was used to compare means, with the Tukey-Kramer post-hoc test to account for multiple pairwise comparisons. To compare the reproducibility of each method, coefficients of variation were determined to describe the percentage of variability in DNA yield relative to the mean for each DNA extraction method. Statistical analyses were conducted in Prism v6 for Windows (GraphPad Software, La Jolla, CA, USA).

### Statistical analyses of sequence data

Paired, two-tailed *t*-tests with Bonferroni adjustment for multiple pairwise comparisons were used to statistically assess differences in the relative abundance of taxon-assigned OTUs between DNA extraction methods. Permutational multivariate analysis of variance (PERMANOVA) was conducted in PERMANOVA+ in PRIMER v6 software and used to unbiasedly assess multivariate data. Values were obtained using type III (partial) sum of squares with 9999 permutations of residuals under a reduced model.

## Results and Discussion

### Influence of extraction method on DNA quantity and quality

**Fig 1:**
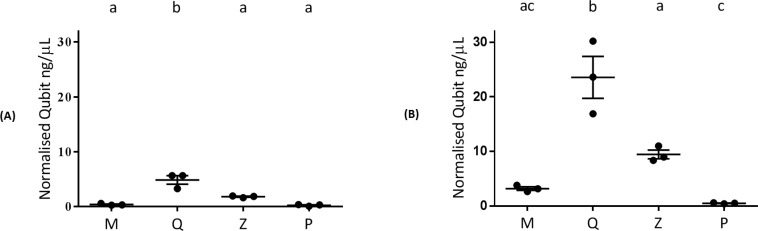
**Normalised DNA yield (ng/**μ**L) from (A) plaque and (B) saliva (mean ± SEM).** M: MoBio PowerSoil^®^ DNA Isolation Kit, Q: QIAamp^®^ DNA Mini Kit, Z: Zymo Bacterial/Fungal DNA Mini Prep^TM^, P: Phenol:chloroform-based DNA isolation. DNA extraction methods not linked by the same letter are significantly different to each other (Tukey-Kramer *p* ≤ 0.05).

Agarose gel images revealed the presence of gDNA for all extraction methods from both plaque and saliva. The Q method yielded the highest concentration of gDNA per 100 μL from both plaque and saliva, while P yielded the lowest in both. Based on normalised Qubit concentrations (mean ± SEM), a significant difference in DNA yield from both plaque and saliva was detected when Q (4.87 ± 0.79 ng/μL) was compared separately to each of the three remaining DNA extraction methods (Fig 1). An enzymatic approach was previously demonstrated to enhance extraction of gDNA from saliva, when compared with mechanical lysis [8]; this is consistent with the highest gDNA yield obtained using the Q method in our study, but does not hold true for the P approach. Therefore, these quantitative results may factor into method selection when gDNA is required for multiple downstream applications. Coefficients of variation (CV) calculated for each method within sample type indicated that all methods were reproducible for DNA yield from both plaque (M = 40%, Q = 28%, Z = 10%, P = 59%) and saliva (M = 18%, Q = 28%, Z = 15%, P = 19%).

DNA purity was assessed across the four methods using the combined average plaque and saliva NanoDrop^®^ values. Overall, the mean (± SEM) M extractions produced the highest A260/280 nm ratios (2.28 ± 0.27), significantly higher than all three other DNA extraction methods: Q (1.72 ± 0.04), Z (1.57 ± 0.03) and P (1.56 ± 0.06), however the M A260/280 nm ratio range was greater than the 1.8–2.0 ratio generally accepted as indicative of pure DNA [27]. Average A260/230 nm ratios indicated the P (1.88 ± 0.14) and Q (1.77 ± 0.14) methods both had significantly less residual carryover than the M (0.92 ± 0.21) and Z (0.72 ± 0.05) methods.

### Bacterial and fungal diversity and composition in plaque is comparable between the four DNA extraction methods

The 12 pooled plaque samples (*n* = 4 methods X *n* = 3 replicates) returned a total of 238,945 unique 16S rRNA gene sequence reads (average length 456 bp). After removal of chimeras (0.9% of unique sequences), *de novo* OTU picking of the remaining sequences returned 325 unique OTUs. Sequencing of ITS1 amplicons from the same 12 plaque samples returned a total of 47,352 unique reads, with an average length of 256 bp. After removal of chimeras (5.4% of unique sequences), *de novo* OTU picking returned 22 unique OTUs.

**Fig 2:**
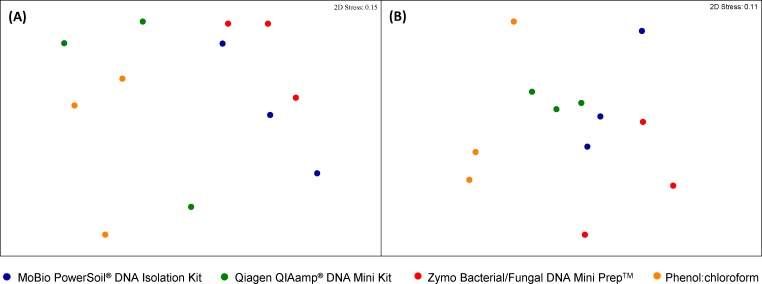
Non-metric multidimensional scaling (MDS) plots comparing relative unweighted UniFrac phylogenetic distances of bacterial communities in (A) plaque and (B) saliva across the four DNA extraction methods.

Multiple pairwise comparisons between DNA extraction methods did not reveal any significant differences in bacterial and fungal species richness and evenness for plaque samples between methods (Table 2). Fungal diversity in plaque was much lower than bacterial diversity, with the mean number of observed OTUs ranging from 2.37–3.07 for fungi and 197–202 for bacteria, for all four methods (Table 2). Furthermore, PERMANOVA analysis of the plaque bacterial community profiles rarefied to 6,000 sequences per sample for unweighted UniFrac revealed that DNA extraction method was not significantly driving differences in the bacterial community profiles (Fig 2A). These results suggest the four DNA extraction methods, with different cell lysis approaches, do not significantly impact on microbial diversity in plaque and agree with previous findings that DNA extraction method is not an influencing factor on plaque community composition [9].

**Table 2:** Alpha diversity measurements (mean ± SEM) for triplicate data from each of the four DNA extraction methods, amplified with 16S rRNA gene primers (A) and ITS1 primers (B).

**(A) 16S rRNA**				
	**Chao1**	**Observed OTUs**	**Shannon diversity**	**Simpson diversity**
**PLAQUE**				
MoBio PowerSoil^®^ DNA Isolation Kit	213 ± 11.33	197 ± 2.29	5.98 ± 0.01	0.97 ± 0.001
Qiagen QIAamp^®^ DNA Mini Kit	221 ± 5.44	201 ± 3.43	6.26 ± 0.02	0.98 ± 0.001
Zymo Bacterial/ Fungal MiniPrep^TM^	220 ± 5.43	202 ± 2.79	6.18 ± 0.03	0.97 ± 0.001
Phenol:chloroform-based isolation	225 ± 2.18	202 ± 1.92	6.32 ± 0.11	0.98 ± 0.003
**SALIVA**				
MoBio PowerSoil^®^ DNA Isolation Kit	153 ± 5.34	120 ± 0.73	4.61 ± 0.02	0.92 ± 0.002
Qiagen QIAamp^®^ DNA Mini Kit	143 ± 6.51	121 ±2.50	4.73 ± 0.06	0.93 ± 0.003
Zymo Bacterial/ Fungal MiniPrep^TM^	162 ± 4.70	122 ± 1.40	4.37 ± 0.05	0.90 ± 0.006
Phenol:chloroform-based isolation	145 ± 5.14	112 ± 2.10	4.53 ± 0.07	0.92 ± 0.005
**(B) ITS1**				
	**Chao1**	**Observed OTUs**	**Shannon diversity**	**Simpson diversity**
**PLAQUE**				
MoBio PowerSoil^®^ DNA Isolation Kit	3.22 ± 0.35	3.07 ± 0.39	0.67 ± 0.16	0.28 ± 0.09
Qiagen QIAamp^®^ DNA Mini Kit	2.37 ± 0.09	2.37 ± 0.09	0.56 ± 0.05	0.23 ± 0.03
Zymo Bacterial/ Fungal MiniPrep^TM^	3.02 ± 0.06	2.90 ± 0.08	0.37 ± 0.03	0.13 ± 0.02
Phenol:chloroform-based isolation	3.27 ± 1.09	3.00 ± 0.71	0.57 ± 0.15	0.23 ± 0.08
**SALIVA**				
MoBio PowerSoil^®^ DNA Isolation Kit	16.16 ± N/A	14.5 ± N/A	1.28 ± N/A	0.40 ± N/A
Qiagen QIAamp^®^ DNA Mini Kit	N/A	N/A	N/A	N/A
Zymo Bacterial/ Fungal MiniPrep^TM^	11.90 ± 0.90	11.90 ± 0.90	2.58 ± 0.24	0.77 ± 0.06
Phenol:chloroform-based isolation	26.98 ± 4.28	23.2 ± 5.20	2.66 ± 0.15	0.70 ± 0.01

**Fig 3:**
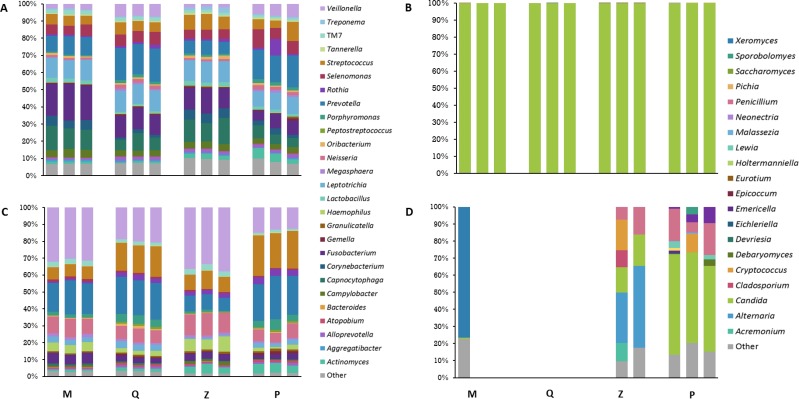
**Taxa plots summarising the relative abundance of taxon-assigned OTUs identified in pooled homogenates for (A) bacterial genera in plaque; (B) fungal genera in plaque; (C) bacterial genera in saliva and (D) fungal genera in saliva.** Each bar represents sequencing from three replicates, rarefied to 6000 sequences per sample for bacterial genera and 959 for fungal genera, with replicates that did not meet these criteria excluded. M: MoBio PowerSoil^®^ DNA Isolation Kit, Q: QIAamp^®^ DNA Mini Kit, Z: Zymo Bacterial/Fungal DNA Mini Prep^TM^, P: Phenol:chloroform-based DNA isolation.

The relative abundance of individual bacterial and fungal taxon-assigned OTUs identified within plaque did not significantly differ across any of the four DNA extraction methods. Sequences assigned to the bacterial genera *Capnocytophaga*, *Fusobacterium*, *Leptotrichia*, *Prevotella*, *Selenomonas* and *Streptococcus* represented the greatest relative abundances in plaque for each method and these genera were recovered at ≥ 5% of the plaque bacterial community in all replicates of each method (Fig 3A). Additionally, *Corynebacterium* comprised > 5% of the plaque community from M replicates and Z methods, while *Veillonella* was found at > 6% in the Q and P methods. Our results are consistent with previous studies that reported these genera as members of the bacterial dental plaque community [2, 28]. Genera present at ≥ 1% were consistently recovered from plaque by all four DNA extraction methods, including the periodontal disease-associated genera *Porphyromonas*, *Tannerella* and *Treponema* [4].

Fungal community profiles from plaque were closely related across the four DNA extraction methods, thus indicating comparable ability of the four approaches to extract fungal DNA from plaque. The plaque mycobiome was almost entirely dominated by *Candida* species, with > 99% of sequences for all methods assigned to this genus (Fig 3B). The closest match (100% sequence identity) through NCBI BLAST indicated that *C*. *albicans* was the dominant species in this genus, across all four methods [29]. Other species also identified within this genus included *C*. *dubliniensis* and *C*. *tropicalis*, although these were less abundant. Given the current lack of available data on the plaque mycobiome, there are no other ITS1 sequencing-based studies against which we can validate our results. Our understanding of the oral mycobiome is thus hindered by the inherent obstacles faced when studying fungal communities. Several factors contribute to a general lack of human oral mycobiome studies, including: the need for well-curated databases comparable to those used for bacterial 16S rRNA gene-based studies, the varying use of primers targeting different regions of the ITS operon between studies, and the unreliable alignment of these targeted ITS sequences. However, an 18S rRNA gene-based study identified *C*. *albicans* as the dominant species in the subgingival plaque of HIV+ patients with low viral loads and high CD4 levels, which may support the current findings [30].

### Effect of DNA extraction method on microbial communities in saliva

Purified reactions of the 12 pooled saliva samples amplified with 16S rRNA gene-targeting primers (*n* = 4 methods X *n* = 3 replicates) returned a total of 360,337 sequences (221,370 unique), with an average length of 459 bp. Chimeras (1.1% of unique reads) were removed and *de novo* OTU picking of the remaining sequences returned 201 unique OTUs. Sequencing of the ITS1 amplicons from the same 12 saliva samples returned a total of 143,832 sequences (19,694 unique reads, average length 287 bp), ranging from 11 to 86,096 sequences per sample. Chimera removal (0.1% of the unique reads) and *de novo* OTU picking returned 92 unique OTUs.

Similarly to plaque, multiple pairwise comparisons of the four DNA extraction methods returned no significant differences in the numbers of observed OTUs in bacterial communities from saliva samples. Method Z yielded the highest number of observed bacterial OTUs (122 ± 1.40), followed by methods Q (121 ± 2.50), M (120 ± 0.73) and finally P (112 ± 2.10). There were no significant differences in Chao1, Shannon or Simpson diversity indices (Table 2). PERMANOVA analysis of the saliva bacterial community profiles rarefied to 6,000 sequences per sample for unweighted UniFrac did not exhibit any significant differences in bacterial community profiles across the four DNA extraction methods (Fig 2B).

The rarefaction threshold excluded several saliva samples from analyses: 3 x Q, 2 x M and 1 x Z; the P method was the only technique to yield adequate numbers of sequences from all three replicates for fungal community diversity analyses. Accordingly, the three P method replicates obtained the highest number of observed fungal OTUs from saliva (23.2 ± 5.20), followed by M (14.5, *n* = 1) and Z (11.9 ± 0.90, *n* = 2). Pairwise comparisons between DNA extraction methods for the average observed fungal OTUs, Chao1, Shannon and Simpson diversity indices did not return any significant differences (Table 2).

*Neisseria*, *Prevotella*, *Streptococcus* and *Veillonella* dominated the bacterial community present in the pooled saliva across all four DNA extraction methods, with the average relative abundance of OTUs assigned to these genera within a method between 7–36% (Fig 3C). This is in agreement with previous studies that identified these genera as dominant members of the salivary bacterial microbiome [31]. Recovery of genera that comprised ≥ 1% of the saliva bacterial community in this study was consistent across all DNA extraction methods.

### The salivary mycobiome is affected by DNA extraction method

The performance of the three commercial DNA extraction kits fell short of the P isolation method when analysing the salivary mycobiome. Various factors could account for this observation. Firstly, given that yeasts and other fungi often have a cell wall which is harder to lyse than bacterial cell walls, the kits utilised in this study may not be optimised for fungal DNA extractions (although the name of at least one of the kits implies otherwise). Additionally, given the fungal load in saliva of healthy individuals is uncertain, the sensitivity of commercial kits for this study may be unsuitable.

Our findings using the P approach indicate that the fungal community in the 12 pooled saliva samples was dominated by members of the genus *Candida*, found at >50% in each replicate. The relative abundance of *Penicillium* was 6–19%, while 14–20% of sequences could not be identified to genus level. *Saccharomyces* and *Malassezia* were only found in P replicates at <10% and < 1%, respectively (Fig 3D). Additionally, this method was not only the sole DNA extraction method to yield adequate sequencing reads for fungal diversity analyses, it was the only method from which *Malassezia* was isolated (present in all saliva replicates). As suggested by Dupuy and colleagues, cell lysis methodology is likely to have a significant effect on identifying *Malassezia* as a fungal community member in saliva [13]. The unique presence of *Malassezia* in the P extractions suggests that this approach was the only one of the four studied to reliably detect this genus in saliva. Data obtained using the P method confirmed most genera of the fungal community common to the only two previous studies of the human salivary mycobiome [11, 13]: *Candida*, *Emericella*, *Lewia*, *Malassezia* and *Saccharomyces* were found in at least two of the three replicates, while *Cryptococcus* was identified from a single replicate (Fig 3D). In contrast to the previous studies, *Cladosporium/Davidiella*, *Fusarium/Gibberella*, *Aureobasidium* and *Epicoccum* were absent in all replicates, although *Epicoccum* was identified in low numbers from two of the three P plaque replicates.

From the Z extractions, fungal sequences assigned to the genus *Alternaria*, a ubiquitous plant pathogen, represented 29% and 48% of the relative abundance of the fungal community in two replicates (Fig 3D). *Candida* was the next most abundant genus, at 15% and 18%. *Cladosporium*, a common indoor and outdoor mould, comprised 10% of sequences in a single replicate but was not found in any other sample that satisfied the rarefication criterion across the four DNA extraction methods. Unassigned sequences made up 9% and 18% of the relative abundance in the two Z replicates.

The single replicate from M to meet our rarefication threshold was largely dominated by *Xeromyces* (77%), a food spoilage mould, which was not present in replicates from the other methods. Additionally, *Candida* and *Penicillium* made up only 0.7% and 0.1% of the respective relative abundances, and sequences to which genus-level identification was not assigned made up 23% (Fig 3D).

### Quality and yield of DNA extracted from *C*. *neoformans* ATCC^®^ 32045^TM^ and *P*. *chrysogenum* ATCC^®^ 10002^TM^ is not significantly different across DNA extraction approaches

Given the variation in salivary mycobiome data between methods, the ability of the four DNA extraction approaches employed here to extract fungal DNA warranted investigation. No significant differences were detected in DNA yield or A260/280 nm and A260/230 nm ratios across the four methods when attempting to extract fungal DNA from *C*. *neoformans* ATCC^®^ 32045^TM^ and *P*. *chrysogenum* ATCC^®^ 10002^TM^ (S1 Table). However, these data suggest that certain extraction approaches are more efficient than others when attempting to extract gDNA from fungal cells.

### Conclusions

Our understanding of the oral microbiome in health and disease is dependent on obtaining an accurate description of the oral microbial community. The many variations in DNA extraction methodology and sequence curation steps that occur between laboratories diminishes consistency and comparability between studies, and may confound results. Here, we examine how different DNA extraction approaches influence our assessment of oral microbial communities by comparing bacterial and fungal diversity and composition using different DNA extraction protocols.

Our findings suggest that the overall quality and yield of gDNA is influenced by DNA extraction approach. The enzymatic approach employed by the Qiagen QIAamp^®^ DNA Mini Kit produced good quality gDNA with significantly greater yield compared to the other three DNA extraction methods. The diversity of bacterial communities in plaque and saliva was largely unaffected by DNA extraction method, with no significant differences in the relative abundance of taxon-assigned OTUs across all four DNA extraction methods. Diversity assessment of the pooled plaque mycobiome also failed to identify discrepancies between DNA extraction methods, however as this community was > 99% *Candida* species, the diversity of our homogenate may have been inadequate for a rigorous comparison of methods.

The phenol:chloroform-based DNA isolation method tested was the only one of the four assessed DNA extraction methods to yield sufficient fungal sequences for analysis from all three saliva replicates. This reinforces the importance of selecting an appropriate DNA extraction method to study oral microbial communities, which should be guided by its ability to produce sufficient and accurate data that address the research question.

## Supporting Information

S1 Table DNA yield and quality measures (mean ± SEM) of triplicate data for extractions from *Cryptococcus neoformans* ATCC^®^ 32045^TM^ and *Penicillium chrysogenum* ATCC^®^ 10002^TM^ across four DNA extraction methods. (DOCX)
