# Supplementary material for: Evaluating the Impact of DNA Extraction Method on the Representation of Human Oral Bacterial and Fungal Communities
Source: PLoS One. 2017 Jan 18;12(1):e0169877. doi: 10.1371/journal.pone.0169877 (PMC5242530; doi:10.1371/journal.pone.0169877)
Supplement: S1 Table — (DOCX) [file pone.0169877.s001.docx]

**S1 Table. DNA yield and quality measures (mean ± SEM) of triplicate data for extractions from *Cryptococcus neoformans* ATCC^®^ 32045^TM^ and *Penicillium chrysogenum* ATCC^®^ 10002^TM^ across four DNA extraction methods.**

|  | ***Cryptococcus neoformans* ATCC^®^ 32045^TM^** | | | ***Penicillium chrysogenum* ATCC^®^ 10002^TM^** | | |
| --- | --- | --- | --- | --- | --- | --- |
|  | **DNA yield (ng/µL)** | **A260/280 nm** | **A260/230 nm** | **DNA yield (ng/µL)** | **A260/280 nm** | **A260/230 nm** |
| MoBio PowerSoil^®^ DNA Isolation Kit | 7.483 ± 2.740 | 1.893 ± 0.066 | 0.500 ± 0.024 | 8.033 ± 3.228 | 1.940 ± 0.034 | 1.140 ± 0.706 |
| Qiagen QIAamp^®^ DNA Mini Kit | 1.167 ± 0.101 | 1.666 ± 0.013 | 0.773 ± 0.408 | 0.667 ± 0.176 | 1.480 ± 0.268 | 0.022 ± 0.459 |
| Zymo Bacterial/ Fungal MiniPrep^TM^ | 3.000 ± 0.477 | 1.979 ± 0.150 | 0.204 ± 0.093 | 4.467 ± 0.900 | 1.910 ± 0.051 | 0.334 ± 0.038 |
| Phenol:chloroform-based isolation | 10.78 ± 2.479 | 1.647 ± 0.068 | 3.213 ± 0.480 | 2.667 ± 0.328 | 1.648 ± 0.044 | 3.338 ± 1.208 |
